# Association between Prurigo Nodularis and Etiologies of Peripheral Neuropathy: Suggesting a Role for Neural Dysregulation in Pathogenesis

**DOI:** 10.3390/medicines7010004

**Published:** 2020-01-08

**Authors:** John-Douglas Matthew Hughes, Taylor E. Woo, Micah Belzberg, Raveena Khanna, Kyle A. Williams, Madan M. Kwatra, Shahzeb Hassan, Shawn G. Kwatra

**Affiliations:** 1 Department of Medicine, Division of Dermatology, University of Calgary, Calgary, AB T2T 5C7, Canada; 2 Cumming School of Medicine, University of Calgary, Calgary, AB T2N 4N1, Canada; tewoo@ucalgary.ca; 3 Department of Dermatology, Johns Hopkins University School of Medicine, Baltimore, MD 21205, USA; mbelzbe@jhu.edu (M.B.); rkhanna8@jhmi.edu (R.K.); kwill184@health.fau.edu (K.A.W.); skwatra1@jhmi.edu (S.G.K.); 4 Department of Anesthesiology, Duke University Medical Center, Durham, NC 27710, USA; madan.kwatra@duke.edu; 5 Feinberg School of Medicine, Northwestern University, Chicago, IL 60611, USA; shahzeb.hassan@northwestern.edu

**Keywords:** prurigo nodularis, nodular prurigo, pruritus, itch, neuropathy

## Abstract

**Background**: Prurigo nodularis (PN) is an intensely pruritic skin condition of considerable morbidity. However, the pathogenesis of PN and its association with underlying neuropathy is unclear. **Objective**: We sought to investigate the association between PN and etiologies of peripheral neuropathy. **Methods**: A cross-sectional analysis of adult patients (≥18-year-old) with PN, AD, and Psoriasis at the Johns Hopkins Health System over a six-year period (January 2013–January 2019) was performed. The strength of association with etiologies of peripheral neuropathy were compared to a control cohort of individuals without PN, as well as those with AD or psoriasis. **Results**: A total of 1122 patients with PN were compared to 10,390 AD patients, 15,056 patients with psoriasis, and a control cohort of 4,949,017 individuals without PN, with respect to 25 comorbidities associated with peripheral neuropathies. **Limitations**: Comparisons between peripheral neuropathies and PN represent associations but are not causal relationships. **Conclusion**: Prurigo nodularis is strongly associated with peripheral neuropathies, suggesting a role for neural dysregulation in pathogenesis.

## 1. Introduction

Prurigo nodularis (PN) is a chronic skin condition characterized by intensely pruritic nodules and hyperkeratotic lesions. Lesions are distributed symmetrically with involvement of extensor surfaces of the extremities. The intractable pruritus of PN is a significant contributor of morbidity for patients and commonly presents in the context of known pruritic conditions [1]. It is associated with other dermatologic conditions, such as atopic dermatitis and other systemic diseases [2]. Examples of systemic diseases associated with PN include liver or kidney dysfunction, hyperthyroidism, metabolic dysfunction, inflammatory processes, psychological factors, and malignancy [3,4,5]. The underlying mechanism of how these conditions contribute to the pathogenesis of PN remains unclear; however, neural dysregulation is thought to play a central role.

Recent studies have studied intraepidermal nerve fiber density (IENFD) as an important contributor to chronic pruritus and in small fiber neuropathies [6,7]. Patients with PN are observed to have decreased IENFD as compared to healthy individuals [7]. Furthermore, there is a significant reduction in IENFD in lesional PN skin, which may be due to prolonged scratching, but also in non-lesional PN [5]. Indeed, the resolution of pruritus is associated with the recovery of dermal nerve fiber density [6]. Cellular changes observed in patients with PN include increased concentrations of substance P and calcitonin gene-related peptide (CGRP) in the nerve fibers of PN patients [8]. Whether these neurophysiological changes are markers of an underlying neuropathies or are a result of the intractable pruritus remains controversial [9]. However, treatments commonly used to treat neuropathic pain have been shown to benefit PN. Agents such as gabapentin and pregabalin have been successfully used to treat patients with PN [10,11,12]. Thalidomide has also been successfully used to treat refractory cases of prurigo nodularis [13,14].

With the evidence surrounding a neural origin of PN unclear, we sought to understand the association between PN and peripheral neuropathies. Furthermore, we conducted a cross-sectional investigation in our health system, comparing patients with PN to individuals with psoriasis, and those with atopic dermatitis.

## 2. Materials and Methods

An association between PN and peripheral neuropathies was investigated by using the Johns Hopkins Health System (JHHS) electronic medical record system, EPIC. The JHHS is a representative collection of approximately 25–30 million individuals located in a 200-mile area within Maryland and its neighboring states and is inclusive of individuals traveling from other countries to the JHHS. Institutional review board approval was not required, as only anonymous, aggregate-level data were used.

A cross-sectional study was performed of patients aged 18 years and older seen at JHHS between 1 January 2013 and 1 January 2019 (n = 4,950,139). EPIC Slicer Dicer was used to collect anonymized aggregate-level data; consequently, IRB approval was waived. SNOMED CT search terms were applied to separately identify patients with PN, atopic dermatitis (AD), psoriasis, or patients without PN. Disease-specific search terms were then applied to identify the prevalence of patients within each group diagnosed with each selected peripheral-neuropathy-related condition. Odds ratios were calculated by comparing the prevalence of patients with PN and each comorbidity to the prevalence of patients in each group with the same comorbidity. Then, *p*-values were calculated, using chi-squared tests with one degree of freedom. To account for multiple comparisons, a Bonferroni-corrected *p*-value was applied. Associations were considered statistically significant if *p* < 0.05.

## 3. Results

### 3.1. Patient Demographics

**Figure 1:**
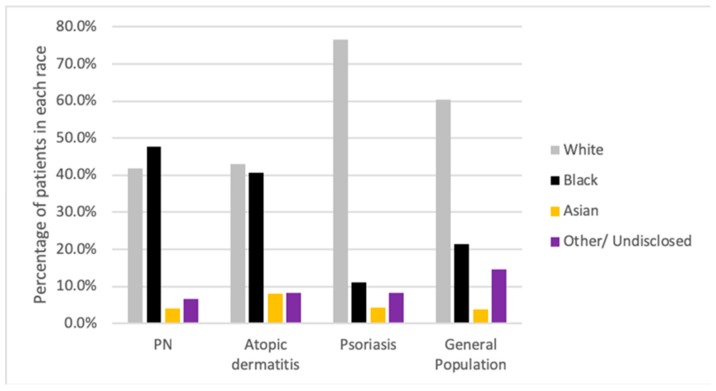
Racial backgrounds of all patients 18 years and older with a diagnosis of prurigo nodularis (PN), atopic dermatitis (AD), or psoriasis, and within the general population who presented to the Johns Hopkins Hospital System between 1 January 2013 and 1 January 2019.

A total of 1122 adult patients with PN, 10,390 AD patients, and 15,056 patients with psoriasis were identified over the six-year period (Figure 1). A control cohort consisting of 4,949,017 individuals without PN was used. In the general population, there was a predominance of Caucasians (61.3%), with African American individuals only representing 21.3% of the patients within the JHHS. Similarly, Caucasians represented over 76.6% of the psoriasis group. In comparison, PN and AD cohorts had differing representative populations. The PN cohort was composed of 41.9% Caucasian and 47.6% African American individuals. Similarly, the AD cohort had 43% and 40.5% of Caucasian and African American patients, respectively.

**Figure 2:**
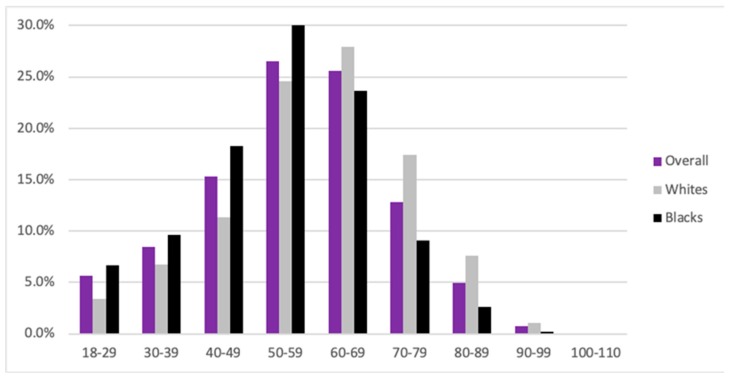
Age distribution of patients with prurigo nodularis (PN) overall, in black and in white patients.

Within the PN study cohort, the proportion of males and females was similar as compared to the general population, with 52% females and 48% males within the PN cohort, and 54.2% females and 45.5% males within the general population. The mean age of PN patients was 56, similar to the general population (56) and the psoriasis cohort (55) (Figure 2). Individuals with atopic dermatitis were comparatively younger, with a mean age of 45. The prevalence of PN increased with age, with the peak prevalence seen between 50 and 59 years of age and composing 26.5% of our cohort. A total of 67.4% of all patients with PN were between 40 and 69 years of age, with individuals between the years of 50 and 59 composing 26.5% of the cohort. 

### 3.2. Association of Prurigo Nodularis with Conditions Known to Feature Peripheral Neuropathies 

Individuals with PN were more likely to have comorbidities associated with peripheral neuropathies as compared to the general population (Table 1). The most common comorbidities seen in patients with PN were statin use (37.34%), diabetes mellitus (26.38%), chronic kidney disease (16.04%), human influenza virus (HIV, 13.37%), metronidazole use (12.39%), and hypothyroidism (11.05%). All the comorbidities analyzed were significantly more common in the PN group, with the exception of small-cell carcinoma (*p* = 0.09). Of the significant comorbidities, borreliosis (Lyme disease) was weakly associated with PN (OR: 3.6, IQR 1.5–8.6, *p* < 0.00001). In contrast, patients with PN were 58 times more likely to have HIV as compared to the general population (OR: 58, IQR 48.8–68.9, *p* < 0.00001). External agents associated with comorbidities of peripheral neuropathies include the use of dapsone, alcohol use, statins, and metronidazole (*p* < 0.00001). Both cobalamin deficiency and folic acid deficiency were associated with peripheral neuropathies in patients with PN. Lastly, inflammatory processes, chronic kidney disease, and neoplasms were associated strongly with PN, including vasculitis (*p* < 0.000001), chronic kidney disease (OR 16.3; 95% CI 13.9–19.2; *p* < 0.00001), and primary malignant neoplasms (*p* < 0.00001). Other conditions, including diabetes mellitus, hyperpituitarism, hypothyroidism, and the presence of myxedema, carpal tunnel syndrome, ulnar nerve entrapment, amyloidosis, vasculitis, and phlebitis, were associated with PN. 

We next examined whether significant differences existed between patients with PN, atopic dermatitis, and psoriasis (Table 1). Among the many differences, patients with PN were more likely to have chronic kidney disease and HIV as compared to both comparator groups (*p* < 000001). However, there were no differences with malignant processes and metabolic conditions, including hyperpituitarism and primary malignant neoplasms, such as small-cell carcinoma and non-small-cell carcinoma. In comparison to atopic dermatitis, no significant differences were observed between carpal tunnel syndrome, use of metronidazole, folic acid deficiency, or hypothyroidism. When comparing the PN cohort to the psoriasis cohort, patients with PN were more likely to have comorbidities associated with peripheral neuropathies, with the exception of myxedema, borreliosis, PMN, folic acid deficiency, and Waldenstrom’s macroglobulinemia.

## 4. Discussion 

The etiology of PN is complex, with both local and systemic disease contributing to the intense pruritus characteristic of the disease [15]. While underlying dermatological conditions, systemic disease, and mood disturbances have been previously associated with PN [16], a limited number of studies have been conducted investigating a neuropathic etiology of PN. Herein, we observed a significant association between PN and comorbidities of peripheral neuropathies. Many of the comorbidities examined, including chronic kidney disease, type-2 diabetes mellitus, and HIV infections, likely contribute both through their association with pruritus and the development of peripheral neuropathies [17]. Furthermore, patients with PN were more likely to have HIV as compared to patients with AD or psoriasis and may be accounted for by the role of HIV as a contributor to severe systemic pruritus [15,18].

Notably, exogenous agents and the association of drug therapies and peripheral neuropathies were observed within our cohort. The use of statins and metronidazole were both more likely to be seen in patients with PN. Evidence suggests that pruritus has been reported in up to 16% of cases associated with statins [19]. Furthermore, both statins and dapsone have been reported in the literature, for their association with peripheral neuropathies [20,21]. Metronidazole is uncommonly associated with pruritus, though it has been associated with the development of peripheral neuropathies in patients exposed to extended high-dose treatment courses [22]. The use of low-dose metronidazole has been reported in several case reports for the management of PN [23]. Lastly, there is an association between patients with PN and depression, anxiety, and greater anxiolytic use [16]. Recent evidence comparing patients with psoriasis and PN found no differences in the psychological profile with respect to the prevalence of anxiety, depression, or somatoform disorders [24]. These findings may account for the increased likelihood of alcohol abuse seen within our PN cohort and its known association with peripheral neuropathies. More importantly, our findings further emphasize the need for increased awareness and consideration of early referral for psychodynamic intervention on behalf of these patients. 

Differences existing between PN, AD, and psoriasis were observed within our study. Interestingly, these differences, although in part likely due to different pathophysiology of these conditions, may also be explained by the difference in burden caused by the pruritus. A recent study examining the burden of pruritus amongst inflammatory dermatoses found that patients with PN were significantly more bothered by evening and nighttime pruritus and reported more significant impairment in quality of life as compared to patients with psoriasis and AD, despite the association of AD and PN [25,26].

We also found that PN was significantly associated with carpal tunnel syndrome, ulnar nerve entrapment, myxedema, hyperpituitarism, amyloidosis, vasculitis, and phlebitis, all of which are novel, to the best of our knowledge, with the exception of amyloidosis [27]. Malignancy was significantly associated with the PN as compared to the general population, likely due to its contributions to pruritus. Recent studies have identified that patients with PN are more likely to have been diagnosed with a malignancy as compared to the general population [28]. However, given the limited number of patients with small-cell carcinoma, it is possible that its lack of association with PN may have represented a statistical anomaly due to limited sample size. 

As a cross-sectional analysis, our comparisons are limited to establishing correlations and not causative relationships between PN and peripheral neuropathies. In addition, the temporality between peripheral neuropathies and exposure to the underlying etiology is not captured by our analysis. It is likely that the pathogenesis of PN and peripheral neuropathies is multifactorial and includes variables not accounted for in our analysis. Lastly, the data are representative of a single center and are, therefore, limited in their generalizability.

## 5. Conclusions

Overall, this study represents a large cross-sectional analysis of PN patients’ to-date, in which an association with peripheral neuropathies is observed. This association with peripheral neuropathies may suggest the importance of neuropathic changes in the pathogenesis of PN. In addition, PN demonstrates distinct differences in comparison to AD and psoriasis, despite their roles as chronic inflammatory pruritic conditions. Clinically, our study underscores an important link between PN and peripheral neuropathies. This may highlight an increased propensity for neural dysregulation in patients with PN. As such, consideration and evaluation of peripheral neuropathies should be considered in patients with PN.

## Figures and Tables

**Table 1:** Absolute number, percentage, odds ratios, and *p*-values of all patients 18 years and older with PN and various comorbid conditions, as compared with those of patients 18 and older with AD, with psoriasis, or within the general population (without PN) who were seen at the JHHS between 1 January 2013 and 1 January 2019.

Comorbidity	PN, n (%)	AD, n (%)	OR (95% CI)	*p*-Value	Psoriasis, n (%)	OR (95% CI)	*p*-Value	Gen Pop, n (%)	OR (95% CI)	*p*-Value
**Peripheral neuropathies**	308(27.45)	1673(16.1)	1.97(1.71–2.27)	<0.002	2759(18.32)	1.69(1.47–1.94)	<0.002	150,325(3.04)	12.08(10.59–13.77)	<0.002
**Focal–multifocal neuropathies**										
Amyloidosis	12(1.07)	31(0.3)	3.61(1.85–7.05)	<0.002	32(0.21)	5.08(2.61–9.88)	<0.002	2234(0.05)	23.94(13.53–42.35)	<0.002
Carpal tunnel syndrome	54(4.81)	397(3.82)	1.27(0.95–1.7)	0.10377	522(3.47)	1.41(1.06–1.88)	0.01893	22,079(0.45)	11.28(8.58–14.83)	<0.002
DM	296(26.38)	1167(11.23)	2.83(2.45–3.28)	<0.002	2648(17.59)	1.68(1.46–1.93)	<0.002	177,323(3.58)	9.64(8.44–11.01)	<0.002
Hyperpituitarism	6(0.53)	37(0.36)	1.5(0.63–3.57)	0.35138	38(0.25)	2.12(0.9–5.04)	0.07978	3268(0.07)	8.14(3.64–18.16)	<0.002
Myxedema	5(0.45)	15(0.14)	3.1(1.12–8.53)	0.02133	27(0.18)	2.49(0.96–6.48)	0.05277	1169(0.02)	18.95(7.86–45.69)	<0.002
Phlebitis	26(2.32)	122(1.17)	2(1.3–3.06)	<0.002	168(1.12)	2.1(1.38–3.19)	<0.002	7395(0.15)	15.85(10.74–23.4)	<0.002
Ulnar nerve entrapment	17(1.52)	74(0.71)	2.14(1.26–3.65)	0.00391	76(0.5)	3.03(1.79–5.15)	<0.002	3546(0.07)	21.46(13.27–34.68)	<0.002
Vasculitis	38(3.39)	208(2)	1.72(1.21–2.44)	0.00231	302(2.01)	1.71(1.22–2.41)	<0.002	13,241(0.27)	13.07(9.45–18.07)	<0.002
**Chronic axonal neuropathies**										
Alcohol abuse	69(6.15)	202(1.94)	3.3(2.5–4.38)	<0.002	462(3.07)	2.07(1.6–2.69)	<0.002	33,073(0.67)	9.74(7.63–12.43)	<0.002
Borreliosis	5(0.45)	63(0.61)	0.73(0.29–1.83)	0.50450	141(0.94)	0.47(0.19–1.16)	0.09351	62,20(0.13)	3.56(1.48–8.57)	0.00250
CKD	180(16.04)	446(4.29)	4.26(3.54–5.13)	<0.002	950(6.31)	2.84(2.39–3.37)	<0.002	57,268(1.16)	16.32(13.91–19.15)	<0.002
Cobalamin deficiency	31(2.76)	204(1.96)	1.42(0.97–2.08)	0.07200	383(2.54)	1.09(0.75–1.58)	0.65366	13,782(0.28)	10.17(7.12–14.55)	<0.002
Dapsone	48(4.28)	132(1.27)	3.47(2.48–4.86)	<0.002	96(0.64)	6.97(4.9–9.9)	<0.002	5420(0.11)	40.76(30.49–54.5)	<0.002
Folic acid deficiency	5(0.45)	14(0.13)	3.32(1.19–9.23)	0.01480	30(0.2)	2.24(0.87–5.79)	0.08659	930(0.02)	23.82(9.87–57.47)	<0.002
HIV infection	150(13.37)	185(1.78)	8.51(6.8–10.66)	<0.002	144(0.96)	15.98(12.6–20.27)	<0.002	13,137(0.27)	57.98(48.78–68.92)	<0.002
Hypothyroidism	124(11.05)	844(8.12)	1.41(1.15–1.72)	<0.002	2066(13.72)	0.78(0.64–0.95)	0.01169	113,182(2.29)	5.31(4.4–6.4)	<0.002
Metronidazole	139(12.39)	1216(11.7)	1.07(0.88–1.29)	0.49875	1098(7.29)	1.8(1.49–2.17)	<0.002	69,631(1.41)	9.91(8.3–11.84)	<0.002
NSCLC	3(0.27)	9(0.09)	3.09(0.84–11.44)	0.07466	33(0.22)	1.22(0.37–3.99)	0.74091	2234(0.05)	5.94(1.91–18.45)	<0.002
Phenytoin	2(0.18)	1(0.01)	18.55(1.68–204.76)	<0.002	2(0.01)	13.44(1.89–95.52)	<0.002	1039(0.02)	8.5(2.12–34.09)	<0.002
PMN	94(8.38)	432(4.16)	2.11(1.67–2.66)	<0.002	894(5.94)	1.45(1.16–1.81)	<0.002	73,237(1.48)	6.09(4.93–7.52)	<0.002
PMN of Lung	6(0.53)	30(0.29)	1.86(0.77–4.47)	0.16086	83(0.55)	0.97(0.42–2.23)	0.94261	7227(0.15)	3.68(1.65–8.2)	<0.002
PMN of Ovary	2(0.18)	4(0.04)	4.64(0.85–25.34)	0.05135	4(0.03)	6.72(1.23–36.73)	0.01090	481(0.01)	18.37(4.58–73.76)	<0.002
Small-cell carcinoma	1(0.09)	4(0.04)	2.32(0.26–20.74)	0.43939	14(0.09)	0.96(0.13–7.3)	0.96736	948(0.02)	4.66(0.65–33.12)	0.09051
Statins	419(37.34)	1999(19.24)	2.5(2.2–2.85)	<0.002	4748(31.54)	1.29(1.14–1.47)	<0.002	361,855(7.31)	7.56(6.69–8.53)	<0.002
Waldenstrom’s disease	1(0.09)	3(0.03)	3.09(0.32–29.72)	0.30358	5(0.03)	2.69(0.31–23.01)	0.34799	293(0.01)	15.07(2.11–107.42)	<0.002

Data are for all patients aged 18 years or older, including 1122 patients with prurigo nodularis (PN), 10,390 patients with AD, 15,056 patients with psoriasis, and 4,949,017 patients in the general population (excluding those with PN). PMN; primary malignant neoplasm; JHHS, Johns Hopkins Health System; CKD, chronic kidney disease; NSCLC, non-small-cell lung cancer; n/a, not applicable; OR, odds ratio; 95% CI, 95% confidence interval.

## References

[1] Kwon C.D., Khanna R., Williams K.A., Kwatra M.M., Kwatra S.G. (2019). Diagnostic Workup and Evaluation of Patients with Prurigo Nodularis. Medicines.

[2] Huang A.H., Canner J.K., Khanna R., Kang S., Kwatra S.G. (2019). Real-world prevalence of prurigo nodularis and burden of associated diseases. J. Investig. Dermatol..

[3] Székely H., Pónyai G., Temesvári E., Berczi L., Hársing J., Kárpáti S., Herszényi L., Tulassay Z., Juhasz M. (2009). Association of collagenous colitis with prurigo nodularis. Eur. J. Gastroenterol. Hepatol..

[4] Payne R., Wilkinson J.D., Mckee P.H., Jurecka W., Black M.M. (1985). Nodular prurigo—A clinicopathological study of 46 patients. Br. J. Dermatol..

[5] Dazzi C., Erma D., Piccinno R., Veraldi S., Caccialanza M. (2011). Psychological factors involved in prurigo nodularis: A pilot study. J. Dermatolog Treat..

[6] Bobko S., Zeidler C., Osada N., Riepe C., Pfleiderer B., Pogatzki-Zahn E., Lvov A., Ständer S. (2016). Intraepidermal Nerve Fibre Density is Decreased in Lesional and Inter-lesional Prurigo Nodularis and Reconstitutes on Healing of Lesions. Acta Derm. Venereol..

[7] Schuhknecht B., Marziniak M., Wissel A., Phan N., Pappai D., Dangelmaier J., Metze D., Ständer S. (2011). Reduced intraepidermal nerve fibre density in lesional and nonlesional prurigo nodularis skin as a potential sign of subclinical cutaneous neuropathy. Br. J. Dermatol..

[8] Vaalasti A., Suomalainen H., Rechardt L. (1989). Calcitonin gene-related peptide immunoreactivity in prurigo nodularis: A comparative study with neurodermatitis circumscripta. Br. J. Dermatol..

[9] Pereira M.P., Pogatzki-Zahn E., Snels C., Vu T.H., Üçeyler N., Loser K., Sommer C., Evers A.W.M., Van Laarhoven A.I.M., Agelopoulos K. (2017). There is no functional small-fibre neuropathy in prurigo nodularis despite neuroanatomical alterations. Exp. Dermatol..

[10] Bharati A., Wilson N.J.E. (2017). Peripheral neuropathy associated with nodular prurigo. Clin. Exp. Dermatol..

[11] Mazza M., Guerriero G., Marano G., Janiri L., Bria P., Mazza S. (2013). Treatment of prurigo nodularis with pregabalin. J. Clin. Pharm Ther..

[12] Huang A.H., Canner J.K., Kang S., Kwatra S.G. (2019). Analysis of real-world treatment patterns in patients with prurigo nodularis. J. Am. Acad. Dermatol..

[13] Aguh C., Kwatra S.G., He A., Okoye G.A. (2018). Thalidomide for the Treatment of Chronic Refractory Prurigo Nodularis. Dermatol. Online J..

[14] Sharma D., Kwatra S.G. (2016). Thalidomide for the treatment of chronic refractory pruritus. J. Am. Acad. Dermatol..

[15] Tarikci N., Kocatürk E., Güngör Ş., Topal I.O., Can P.Ü., Singer R. (2015). Pruritus in Systemic Diseases: A Review of Etiological Factors and New Treatment Modalities. Sci. World J..

[16] Jørgensen K.M., Egeberg A., Gislason G.H., Skov L., Thyssen J.P. (2017). Anxiety, depression and suicide in patients with prurigo nodularis. J. Eur. Acad. Dermatol. Venereol..

[17] Boozalis E., Tang O., Patel S., Semenov Y.R., Pereira M.P., Stander S., Kang S., Kwatra S.G. (2018). Ethnic differences and comorbidities of 909 prurigo nodularis patients. J. Am. Acad. Dermatol..

[18] Matthews S.N., Cockerell C.J. (1998). Prurigo nodularis in, H.I.V-infected individuals. Int J. Dermatol..

[19] Reich A., Ständer S., Szepietowski J.C. (2009). Drug-induced pruritus: A review. Acta Derm.-Venereol..

[20] Tierney E.F., Thurman D.J., Beckles G.L., Cadwell B.L. (2012). Association of statin use with peripheral neuropathy in the, U.S. population 40 years of age or older. J. Diabetes.

[21] Waldinger T.P., Siegle R.J., Weber W., Voorhees J.J. (1984). Dapsone-Induced Peripheral Neuropathy. Arch. Dermatol..

[22] Goolsby T.A., Jakeman B., Gaynes R.P. (2018). Clinical relevance of metronidazole and peripheral neuropathy: A systematic review of the literature. Int J. Antimicrob. Agents.

[23] Spring P., Gschwind I., Gilliet M. (2014). Prurigo nodularis: Retrospective study of 13 cases managed with methotrexate. Clin. Exp. Dermatol..

[24] Schneider G., Hockmann J., Ständer S., Luger T.A., Heuft G. (2006). Psychological factors in prurigo nodularis in comparison with psoriasis vulgaris: Results of a case-control study. Br. J. Dermatol..

[25] Steinke S., Zeidler C., Riepe C., Bruland P., Soto-Rey I., Storck M., Augustin M., Bobko S., Garcovich S., Legat F.J. (2018). Humanistic burden of chronic pruritus in patients with inflammatory dermatoses: Results of the European Academy of Dermatology and Venereology Network on Assessment of Severity and Burden of Pruritus (PruNet) cross-sectional trial. J. Am. Acad. Dermatol..

[26] Iking A., Grundmann S., Chatzigeorgakidis E., Phan N.Q., Klein D., Ständer S. (2013). Prurigo as a symptom of atopic and non-atopic diseases: Aetiological survey in a consecutive cohort of 108 patients. J. Eur. Acad. Dermatol. Venereol..

[27] Baykal C., Ozkaya-Bayazit E., Gökdemir G., Diz Küçükkaya R. (2000). The combined occurrence of macular amyloidosis and prurigo nodularis. Eur. J. Dermatol..

[28] Larson V.A., Tang O., Stander S., Miller L.S., Kang S., Kwatra S.G. (2019). Association between prurigo nodularis and malignancy in middle-aged adults. J. Am. Acad. Dermatol..

